# Concordance Between the Occlusal Contacts Record Obtained Using an Intraoral Scanner and Carbon Paper

**DOI:** 10.1111/ocr.70088

**Published:** 2026-01-28

**Authors:** Victor França Didier, Victor de Miranda Ladewig, Jessica Quereza de Freitas, Paula Vanessa Pedron Oltramari, Thaís Maria Freire Fernandes, Marcio Rodrigues de Almeida, Renata Rodrigues de Almeida Pedrin, José Fernando Castanha Henriques, Ana Cláudia de Castro Ferreira Conti

**Affiliations:** ^1^ Orthodontic Department Unopar Londrina PR Brazil; ^2^ Orthodontic Department Uniderp Campo Grande MS Brazil; ^3^ Department of Orthodontics, Bauru Dental School University of São Paulo Bauru SP Brazil

**Keywords:** intraoral scanner, occlusal contacts, orthodontics

## Abstract

**Objectives:**

To evaluate the reliability of the registration of occlusal contacts through intraoral scanning in comparison with those obtained with the aid of carbon paper.

**Materials and Methods:**

The occlusal registration was obtained at the beginning of the orthodontic treatment of 35 patients (23 men and 12 women), aged 15 to 30 years. All patients were scanned with iTero Element (Align Technology, CA, USA), occlusal records were also performed with carbon paper (AccuFilm – 8 μm, USA) and recorded in occlusograms. To verify the agreement between the two methods, the percentages of agreement and disagreement and kappa statistics were applied.

**Results:**

There was poor agreement between occlusal contacts recorded by intraoral scanning and those obtained with carbon paper in most of the sample (Kappa value 0.07 to 0.20). Most contacts were registered in the posterior region. It seems that the contacts registered as intense in the iTero, correspond to the contacts with the carbon paper.

**Conclusions:**

The occlusal records by means of scanning and carbon paper presented poor agreement, but the association of both methods is indicated for the correct registration of the occlusion.

## 1 Introduction

Intraoral scanners are a reality in the dentist's clinical activity, being widely used in restorative and prosthetic dentistry, as well as for orthodontic diagnosis, treatment planning and treatment monitoring [1, 2]. Their use has increased substantially among clinicians, and they are well accepted by patients due to greater comfort and overall satisfaction [2, 3, 4].

There are studies on the accuracy and effectiveness of scans, as well as comparisons of the mapping performed by intraoral scanners [3]. These findings support the notion that modern devices, even when produced by different manufacturers, demonstrate comparable levels of precision. However, further efforts are required to expand and optimize the clinical applications of this technology [3, 5, 6].

However, to expand scanning application methods, it is essential to ensure that the information obtained is reliable, including the occlusal relationship of the arches and contact points. Several studies have focused on validating this occlusal relationship of the arches in a virtual environment; however, further investigations are needed to compare the relationship obtained in scanning with that observed in patients in vivo [6, 7, 8, 9].

In recent years, the demand for aligner therapy and its associated planning has increased considerably, directly contributing to a higher number of intraoral scans performed for both treatment planning and monitoring [3, 10, 11, 12]. Based on the individualized treatment plan, ClinCheck (Align Technology's orthodontic planning software) displays the planned tooth movements and predicts treatment outcomes through a virtual model. The clinical outcome of aligner therapy is expected to correspond to this final virtual position [13].

Even with all this technology, it is observed that there is a tendency for molar intrusion in 74% of patients [12, 14, 15, 16]. Therefore, having the ability to accurately read the occlusal relationship in the scan will enable predicting with greater precision the areas where there is or isn't contact, thus minimizing the tendency for posterior open bite or any other occlusal misalignment, improving case finalization and allowing, moreover, the closure of treatments with simultaneous contacts on all posterior teeth bilaterally [12, 17].

Recent investigations have increasingly explored the reliability of digital systems for occlusal contact registration. Reich et al. showed that digital systems such as T‐Scan tend to underestimate the number of occlusal contacts compared to conventional methods, although they provide higher reproducibility, especially in anterior regions [18]. In contrast, Wei et al. introduced a three‐dimensional evaluation protocol and demonstrated that intraoral scanning achieved a consistency rate of occlusal contact regions nearly three times higher than conventional impressions, in addition to showing significantly improved occlusal tightness, although virtual intersections were occasionally detected [19]. Complementing these findings, Rovira‐Lastra et al. reported that silicone transillumination achieved reproducibility rates above 85%, while articulating films and digital systems such as virtual occlusion and T‐Scan displayed only moderate validity and reproducibility [20]. These limitations highlight the need for further research to clarify the concordance between digital and conventional methods under standardized conditions.

Therefore, the objective of this study was to determine the level of agreement between occlusal contacts recorded by intraoral scanning and those obtained with carbon paper, considering both the number of contacts and their distribution across tooth areas.

## 2 Materials and Methods

The sample of this retrospective study was obtained from a database from the University of Northern Paraná (Unopar), and occlusal records of 35 patients (12 women and 23 men) were used. All participants provided valid consent for their complete records to be used for this study. This study was approved by the Research Ethics Committee (CEP) of the University of Northern Paraná (Unopar), process number 3384099.

The kappa statistic was used to assess the precision of the occlusal contact records, with a mean kappa value of 0.14 and a 95% confidence level. The margin of error for the kappa estimates was ±0.17 for canines and first molars (*n* = 140) and ±0.12 for incisors and premolars (*n* = 280) [21]. Accordingly, for the larger sample, the true kappa value is expected to range from 0.02 to 0.26 with 95% confidence. Each tooth from the 35 patients was considered an independent sampling unit, resulting in a total of 840 teeth, thereby enhancing the precision of the agreement estimates.

The inclusion criteria were patients with Class I malocclusion, aged 15 to 30 years, with moderate crowding (3‐5 mm) and without tooth extraction. The exclusion criteria were absence of permanent teeth, previous open bite, crossbite or previous orthodontic treatment.

Patients were instructed to perform mandibular movements (opening and closing into maximum habitual intercuspation with maximum intensity) while seated in a dental chair at a 45° angle. All occlusal surfaces were dried with an air jet before recording, initially with carbon paper and then with intraoral scanning.

**FIGURE 1:**
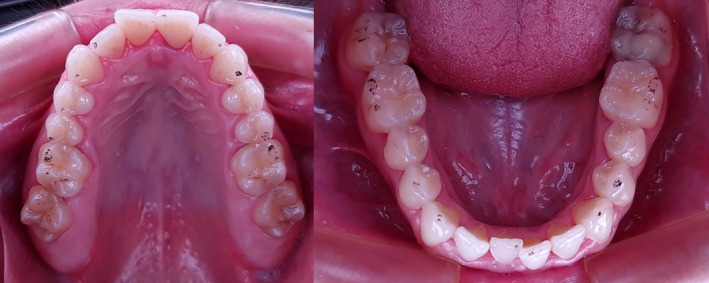
Upper and lower arches showing the occlusal contacts registration with the carbon paper.

The records obtained with the aid of carbon paper were carried out by a single professional, using carbon paper (articulating foil) with a thickness of eight microns (8 μm) (AccuFilm, USA) (Figure 1). After obtaining the points, the contacts were transferred to an occlusogram for further evaluation.

**FIGURE 2:**
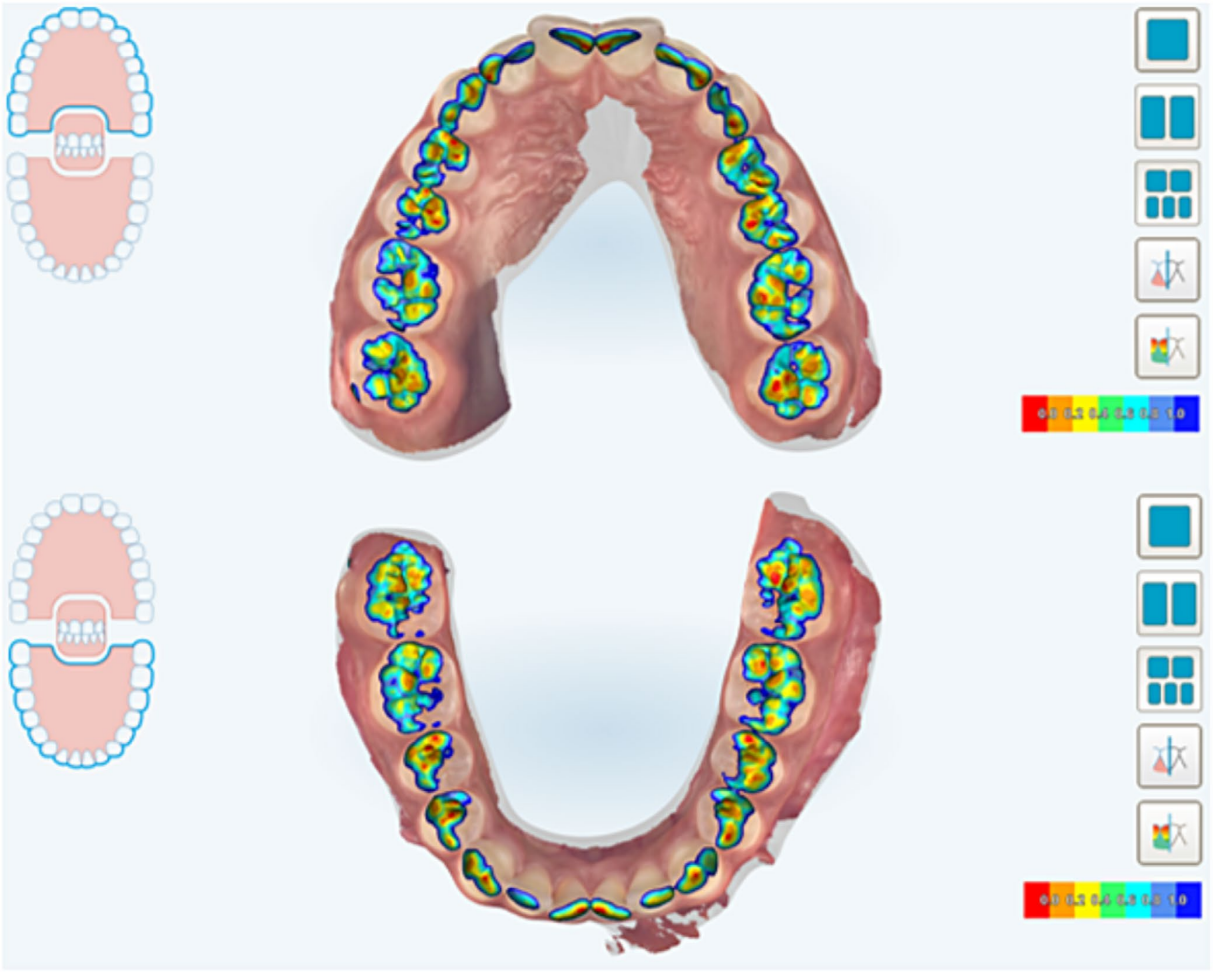
Contact points generated from intraoral scanning represented by means of a colour scale that varies from red to dark blue, according to the distance between the occlusal surfaces.

The iTero Element 2 Scanner (Align Technology, Santa Clara, USA) was used for intraoral scanning; patient registration was carried out by 2 other professionals, previously trained to perform the same scanning path, and obtain the image through full‐arch scanning. Intraoral scanning generates a digital model in which the contact points are represented using a colour scale that varies from red to dark blue, according to the distance between the occlusal surfaces (Figure 2). These contacts were transferred to an occlusogram with the aid of coloured pencils, respecting the colour scale and considering only the colours red and orange as contacts. This colour range was determined because the variation according to the company itself would be from 0.0 mm to 0.1 mm (red) and 0.1 mm to 0.2 mm (orange).

The contacts were evaluated in the first molars, first and second premolars, canines, lateral and central incisors, on the left and right sides, in the upper and lower arches (Table 1).

**TABLE 1:** Description of the teeth and their respective occlusal contact areas.

First molar	Mesiobuccal cusp (MBC)	Distobuccal cusp (DBC)	Mesiolingual cusp (MLC)	Distolingual cusp (DLC)	Central groove (CG)
First and second premolar	Buccal cusp (BC)	Lingual cusp (LC)	Central groove (CG)	Mesial marginal ridge (MMR)	Distal marginal ridge (DMR)
Canine	Mesial lingual surface (MLS)	Distal lingual surface (DLS)	Tip of cusp (TC)		
Central and lateral incisors	Mesial buccal/lingual surface (MS)	Distal buccal/lingual surface (DS)	Incisal edge (IE)		

### 2.1 Statistical Analyses

All statistical procedures were performed using SPSS version 28. Data were described by relative frequency (%). The definition of contact by the scanner was evaluated in three forms: (1) when red or orange colours occurred; (2) when only the red colour occurred; and (3) when only the orange colour occurred.

To check the agreement between the two methods, the percentages of agreement and disagreement and the kappa statistic were applied, with the colours red and orange being considered contact for the scanner. And these results were interpreted according to the kappa value of Landis & Koch [22].

## 3 Results

According to Table 2 (descriptive data) through registration with occlusal carbon, 917 contact points were obtained, while during scanning, 1548 contact regions were obtained in total, 745 red (48.1%) and 803 orange (51.9%). In incisors, canines and premolars, the prevalence of red regions was higher (60.3%, 54.8% and 52.1%, respectively), whereas in molars, orange contacts predominated (69.1%).

**TABLE 2:** Proportion of red and orange markings by the scanner method and total markings for carbon.

Tooth	Scanning					Carbon	
	Red		Orange		Total *n*%	Total *n*	%
	*n*	%	*n*	%			
Incisors	211	60.3	139	39.7	350	159	17.33
Canines	86	54.8	71	45.2	157	133	14.5
Premolars	311	52.1	286	47.9	597	365	39.8
First molar	137	30.9	307	69.1	444	260	28.35
Total *n*	745	48.1	803	51.9	1548	917	100

When comparing the number of contacts, more occlusal contacts were recorded in the premolar region than in the molar region, both for carbon (365 in premolars; 260 in molars) and for scanning (Table 2). Comparing the methods, more contacts were obtained in carbon than in the red regions for canine teeth, premolars and molars (Table 2).

Analyzing the results overall, low or no agreement was found for most occlusal records between the two methods, whether comparing the sum of the red and orange contacts from the scan with the carbon contacts, or considering only the red contacts. According to the kappa statistics, most tooth surfaces exhibited poor agreement between the carbon paper and the intraoral scanner in recording occlusal contacts (Kappa value 0.07 to 0.20). Only the Lingual Cusp (LC) of premolars (Kappa value 0.38) and the Mesiolingual Cusp (MLC) of first molars (Kappa value 0.27) showing slight agreement (Table 3).

**TABLE 3:** Percentage distribution of agreements and disagreements between Carbon (C) and Scanner (S), and kappa statistics, for central and lateral incisors, canines, premolars and molars. Considering contact by the scanner when red or orange.

Tooth	Area (Total)	Agreement		Disagreement		Total of C = Yes	Total of S = Yes	Total		
		C and S	C and S	C = Yes	S = Yes			Agreement	Disagreement	kappa
		Yes	No	S = No	C = No					
Incisors	MS	3.6	58.9	1.8	35.7	5.4	39.3	62.5	37.5	0.07
	DS	9.3	54.6	2.5	33.6	11.8	42.9	63.9	36.1	0.19
	IE	21.4	39.6	17.5	21.4	38.9	42.9	61.1	38.9	0.20
Canines	MLS	2.9	64.3	2.9	30.0	5.7	32.9	67.1	32.9	0.06
	DLS	8.6	58.3	6.5	26.6	15.1	35.3	66.9	33.1	0.17
	TC	33.6	17.9	37.9	10.7	71.4	44.3	51.4	48.6	0.07
Premolars	BC	47.5	14.3	9.3	28.9	56.8	76.4	61.8	38.2	0.18
	LC	24.6	45.4	8.6	21.4	33.2	46.1	70.0	30.0	0.38
	CG	1.1	83.6	10.0	5.4	11.1	6.4	84.6	15.4	0.04
	MMR	6.4	57.9	2.9	32.9	9.3	39.3	64.3	35.7	0.13
	DMR	10.7	48.6	6.4	34.3	17.1	45.0	59.3	40.7	0.13
Molars	MBC	29.3	22.9	3.6	44.3	32.9	73.6	52.1	47.9	0.18
	DBC	43.6	15.0	5.7	35.7	49.3	79.3	58.6	41.4	0.18
	MLC	28.6	34.3	10.7	26.4	39.3	55.0	62.9	37.1	0.27
	DLC	36.4	14.3	6.4	42.9	42.9	79.3	50.7	49.3	0.09
	CG	7.9	60.0	10.0	22.1	17.9	30.0	67.9	32.1	0.13

Considering only the red contact regions for comparison with carbon paper, the same trend of poor agreement was observed. The highest values of agreement, classified as slight by kappa, were found only at the Distal Buccal/Lingual Surface (DS) side of the incisor (Kappa value 0.21) and on the Buccal Cusp (BC) and Lingual Cusp (LC) of premolars, 0.21 and 0.26 respectively. For the remaining surfaces, the agreement ranged from poor to none (Table 4).

**TABLE 4:** Percentage distribution of agreements and disagreements between Carbon (C) and Scanner (S), and kappa statistics, for central and lateral incisors, canines, premolars and molars. Considering contact by the scanner only when red.

Tooth	Area (Total)	Agreement		Disagreement		Total of C = Yes	Total of S = Yes	Total		
		C and S	C and S	C = Yes	S = Yes			Agreement	Disagreement	Kappa
		Yes	No	S = No	C = No					
Incisors	MS	2.5	75.7	2.9	18.9	5.4	21.4	78.2	21.8	0.11
	DS	6.1	70.0	5.7	18.2	11.8	24.3	76.1	23.9	0.21
	IE	15.7	47.1	23.2	13.9	38.9	29.6	62.9	37.1	0.18
Canines	MLS	2.1	80.0	3.6	14.3	5.7	16.4	82.1	17.9	0.12
	DLS	5.0	72.7	10.1	12.2	15.1	17.3	77.7	22.3	0.18
	TC	22.1	22.9	49.3	5.7	71.4	27.9	45.0	55.0	0.08
Premolars	BC	28.6	31.1	28.2	12.1	56.8	40.7	59.6	40.4	0.21
	LC	14.3	54.6	18.9	12.1	33.2	26.4	68.9	31.1	0.26
	CG	0.0	87.5	11.1	1.4	11.1	1.4	87.5	12.5	−0.03
	MMR	2.9	73.2	6.4	17.5	9.3	20.4	76.1	23.9	0.07
	DMR	4.3	65.0	12.9	17.9	17.1	22.1	69.3	30.7	0.03
Molars	MBC	10.0	52.1	22.9	15.0	32.9	25.0	62.1	37.9	0.09
	DBC	15.0	44.3	34.3	6.4	49.3	21.4	59.3	40.7	0.18
	MLC	11.4	51.4	27.9	9.3	39.3	20.7	62.9	37.1	0.15
	DLC	13.6	44.3	29.3	12.9	42.9	26.4	57.9	42.1	0.10
	CG	2.1	80.0	15.7	2.1	17.9	4.3	82.1	17.9	0.13

## 4 Discussion

Intraoral scanning systems and their associated software are widely used in clinical practice. Virtual casts aligned with virtual interocclusal records should permit the accurate identification and quantification of any regions of contact between the meshes representing the virtual arches [23]. The findings of the present study indicated that occlusal registration with carbon paper was different from that obtained with intraoral scanning.

When considering the concordance of the carbon marking with the sum of the red and orange contacts of the scanner, there was a greater registration through the scanning (around 70%) the number of contacts obtained with carbon paper. In contrast, when only red contact regions were considered, fewer contacts were identified in the scans than with carbon paper. These results reaffirm the difference between the real and the virtual and raising the hypothesis that perhaps the orange mapping of the scan (representation of occlusal proximity between 0.1 mm and 0.2 mm, Figure 2) is a lack of contact [24, 25, 26].

Both methods showed a greater number of occlusal contacts in the posterior region (Table 2), which is expected for a stable occlusion [17]. However, with carbon paper, more contacts were recorded in the region of canines (133), premolars (365) and molars (260), than that considered for each dental type in the red marking of the scan (respectively 86; 311; 137). This fact can be justified by the presence of the occlusal paper, which despite being the thinnest as recommended, it is still an interposed object [24]. Additionally, studies reinforce the limited effectiveness of intraoral scanning for recording the occlusal relationship in full arch [24, 25, 27]. From a prosthodontic perspective, patients are capable of perceiving occlusal discrepancies as small as 10 μm, which corresponds to the sensitivity threshold detected with articulating paper (8 μm). In contrast, the iTero scanner defines red contacts at distances up to 100 μm (the minimum distance provided by the scanner), which indicates a potential ten‐fold reduction in accuracy compared to conventional methods.

Kappa analysis revealed poor agreement between occlusal registration methods across most tooth surfaces (Table 3), with the exception of two tooth surfaces showing slight agreement when red and orange regions were considered as contacts (LC in premolars and MLC in first molars), and three regions showing slight agreement when only red regions were considered (DS of incisor and BC and LC of premolars). There is an erroneous correlation of considering the ClinCheck red contacts as excessive since 48.1% of the intense contacts were clinically presented as ideal contacts, which would allow us to consider the hypothesis that the early removal of the red contacts from the simulation may be a factor that causes an unexpected “overcorrection” of the occlusion. Furthermore, it enhances the deepening of the bite in treatments with aligners, as an unnecessary intrusion of the teeth is caused [12, 16, 25]. In agreement with these results, another study states that scanning tends to make closer contacts, generating an overcorrection in the reading of the occlusal register [23]. An intermediate category between red and orange contact on iTero scanning and an intermediate category between red and green on Clincheck, may be necessary to achieve these differences.

The determination of the gold standard as a reference for occlusal registration has not yet been defined in the literature, carbon paper is used routinely, but it has its limitations and operator variability [28]. The use of transillumination records contacts in separations greater than 0.35mm and the use of technologies such as T‐Scan, despite recording the intensity of contacts, may interfere with the vertical dimension of occlusion and muscle function [9, 29]. In the present study, only one scanner model (iTero Element 2 Scanner, Align Technology, Santa Clara, USA) was employed in this study. Thus, the results cannot be generalized to other intraoral scanning systems, and this represents an important limitation. Another limitation is that teeth with restorative materials were not excluded from the sample. However, as the study involved a young population, most teeth were sound or presented only small composite resin restorations. Although such restorations may reflect light differently and potentially influence digital image construction, their impact on the overall accuracy of the records is likely minimal. Furthermore, due to the retrospective design, it was not possible to perform intra‐examiner reliability tests, as the patients had already been treated, making it impossible to register occlusal records twice. This limitation should be considered in future prospective studies.

Based on the findings of the present study, establishing the occlusal relationship exclusively using iTero scan data cannot be recommended. Therefore, further studies are required to elucidate and standardize occlusal registration methods, as well as to define a reliable and clinically meaningful relationship between occlusal registration and digital technologies.

## 5 Conclusion

- Occlusal registration obtained by intraoral scanning and carbon paper demonstrated poor agreement.
- Most occlusal contacts were located in the posterior region.
- These findings may suggest that considering both methods in clinical practice may enhance the reliability of occlusal registration.

## Authors’ Contributions

**Victor França Didier:** Conceptualization, Methodology, Validation, Investigation, Writing, Original Draft, Formal Analysis. **Victor de Miranda Ladewig:** Visualization and Writing, Review & Editing. **Jessica Quereza de Freitas:** Visualization and Writing, Review & Editing. **Paula Vanessa Pedron Oltramari:** Visualization and Writing, Review & Editing. **Thaís Maria Freire Fernandes:** Visualization and Writing, Review & Editing. **Marcio Rodrigues de Almeida:** Visualization and Writing, Review & Editing. **Renata Rodrigues de Almeida‐Pedrin:** Visualization and Writing, Review & Editing. **José Fernando Castanha Henriques:** Writing, Review & Editing, Supervision. **Ana Cláudia de Castro Ferreira Conti:** Resources, Project Administration, Funding Acquisition, Review & Editing, Supervision.

## Funding

The authors have nothing to report.

## Ethics Statement

This study was approved by the Research Ethics Committee (CEP) of the University of Northern Paraná (Unopar), process number: 3.384.099.

## Conflicts of Interest

The authors declare no conflicts of interest.

## Data Availability

The data that support the findings of this study are available on request from the corresponding author. The data are not publicly available due to privacy or ethical restrictions.
